# Relationships of catch-per-unit-effort metrics with abundance vary depending on sampling method and population trajectory

**DOI:** 10.1371/journal.pone.0233444

**Published:** 2020-05-21

**Authors:** Maximilian L. Allen, Nathan M. Roberts, Javan M. Bauder

**Affiliations:** 1 Illinois Natural History Survey, University of Illinois, Champaign, Illinois, United States of America; 2 Wisconsin Department of Natural Resources, Rhinelander, Wisconsin, United States of America; National Oceanic and Atmospheric Administration, UNITED STATES

## Abstract

Catch-per-unit-effort (CPUE) is often used to monitor wildlife populations and to develop statistical population models. Animals caught and released are often not included in CPUE metrics and their inclusion may create more accurate indices of abundance. We used 21 years of detailed harvest records for bobcat (*Lynx rufus*) in Wisconsin, U.S.A., to calculate CPUE and ‘actual CPUE’ (ACPUE; including animals caught and released) from bobcat hunters and trappers. We calibrated these metrics to an independent estimate of bobcat abundance and attempted to create simple but effective models to estimate CPUE and ACPUE using harvest success data (i.e., bobcats harvested/available permits). CPUE showed virtually no relationship with bobcat abundance across all years, but both CPUE and ACPUE had stronger, non-linear, and negative relationships with abundance during the periods when the population was decreasing. Annual harvest success strongly predicted composite ACPUE and CPUE from hunters and trappers and hunter ACPUE and CPUE but was a poorer predictor of trapper ACPUE and CPUE. The non-linear, and sometimes weak, relationships with bobcat abundance likely reflect the increasing selectivity of bobcat hunters for trophy animals. Studies calibrating per-unit-effort metrics against abundance should account for population trajectories and different harvest methods (e.g., hunting and trapping). Our results also highlight the potential for estimating per-unit-effort metrics from relatively simple and inexpensive data sources and we encourage additional research into the use of per-unit-effort metrics for population estimation.

## Introduction

Quantifying and estimating trends in wildlife abundance is critical for wildlife management and conservation, but many species are cryptic leading to innate difficulties in estimating abundance [1, 2]. As a result, population indices, including harvest-based indices, are often used as surrogate indices for wildlife populations [1, 3]. Harvest records may span multiple decades, and may provide the only long-term data source for certain species or populations in a given management unit [4–6]. These records also form an important component for integrating age-at-harvest data, harvest effort, and ancillary data (e.g., radio telemetry data) within population models to estimate abundance over time [2, 7, 8].

Harvest effort is generally represented as a form of catch-per-unit-effort (CPUE) [7, 8], often derived as catch per number of days spent hunting. Because CPUE is often easier to obtain (i.e., through post-harvest questionnaires) than direct estimates of abundance or density and may vary proportionally with abundance/density, it may hold potential as a metric with which to monitor wildlife populations directly. As with other population indices, it is important to calibrate CPUE metrics prior to using those metrics to evaluate population status and trends [1]. More importantly, the strength and direction of the relationship between CPUE and abundance/density in harvested populations of terrestrial mammals can vary and does not always correlate proportionally with abundance [9], which can be a problem when using CPUE to monitor wildlife populations. A lack of proportional relationship between CPUE and abundance/density may be a function of factors affecting harvest effort, including population status, socio-economic factors, and environmental conditions [10–12]. These factors may also have different effects on CPUE for hunters and trappers, even when targeting the same species [e.g., 13].

Another factor affecting hunter/trapper effort is selectivity for the harvest of individuals with certain traits [e.g., large antler or body size, 11, 13–15]. For example, deer hunters, when searching for a “trophy” animal, may pass on harvesting multiple different individuals [e.g., 16]. Such selectivity could directly affect CPUE metrics if hunters/trappers forgo the harvest of multiple encountered animals until they encounter one with desired traits [e.g., 16], especially for species with restricted harvest limits [13]. In such cases, CPUE may not be as informative as a per-unit-effort metric which takes into account the total number of animals captured including those caught and released (hereafter termed actual-catch-per-unit-effort; ACPUE). It is therefore important to consider whether ACPUE may be a more useful index than CPUE, as well as understand the factors influencing variation in CPUE and ACPUE.

We compared the relationships between CPUE, ACPUE, and abundance using 21 years of bobcat (*Lynx rufus*) harvest data from Wisconsin, U.S.A. Bobcats are territorial, cryptic mesocarnivores [17, 18] that are frequently harvested as furbearers throughout North America [19, 20]. Within Wisconsin, most bobcat are harvested using trained hounds (hereafter hunters) and trapping (hereafter trappers), with small amounts of harvest from calling, unaided hunting, and incidental trapping [13]. Once an animal is caught, it can be harvested or released (released from a trap or ending the hunt). Our first objective was to determine if successful hunters/trappers spent more days in the field, used more traps, and released more bobcats. We hypothesized that successful hunters/trappers would hunt/trap for more days than unsuccessful hunters, and that successful hunters and trappers would release more bobcats than unsuccessful ones. Our second objective was to compare and characterize the relationships of CPUE and ACPUE for hunters and trappers to independent estimates of bobcat abundance and estimate the strength of those relationships. Finally, because collecting data on hunter/trapper effort may still require a substantial investment in resources by wildlife management agencies, we attempted to create an index that is easier to collect for effectively estimating CPUE and ACPUE metrics with minimal resources for inclusion in population models.

## Materials and methods

### Ethics statement

All data used in this study were collected by the Wisconsin Department of Natural Resources from legally harvested animals and as such there was no use of live animals in this study. The authors did not handle, collect or kill any animals for this study. The authors observed all ethical guidelines from the Wisconsin Department of Natural Resources and the University of Illinois, as well as all national and international guidelines.

### Study area

Bobcat hunting and trapping in Wisconsin is divided into two zones (north and south) with the boundary between zones corresponding to United States Highway 64 [13]. We used data from bobcats harvested from 1973–2013 in Wisconsin’s northern zone, because harvest for bobcats in Wisconsin’s southern zone only began in 2014 with a limited harvest. The northern zone makes up approximately the northern third of the state which is dominated by the North Central Forest, Northern Highland, and Forest Transition ecological landscapes [21]. Primary habitats include a diversity of mesic and upland hardwood, mixed hardwood-conifer, and conifer forests as well as forested and non-forested wetlands. Anthropogenic habitats in the form of agriculture and urban development are less prevalent than in the southern zone.

### Data collection

Bobcat harvest has become more regulated over the past century in Wisconsin [22], with the current system being a lottery quota system where hunters/trappers gain preference points for each year they apply but do not earn a harvest permit [22]. Since 1973, the Wisconsin Department of Natural Resources (WDNR) has required bobcat hunters to register every harvested bobcat with WDNR regulatory personnel. The sex of each registered harvested bobcat was recorded and verified, a tooth extracted for aging using cementum annuli, and the uteri were examined for placental scars. The number of harvest permits issued has decreased over the last few decades and corresponded to an increase in harvest success [23, 24], while the number of bobcat hunters has also increased relative to the number of bobcat trappers [13]. We estimated annual abundance using a sex-age-kill model [25, 26] with data on sex- and age-specific harvest, sex-age composition, and age-specific reproductive rates [27].

We sent post-season questionnaire surveys annually from 1993–2013 (Supporting Information 1) to every bobcat hunter/trapper who received a harvest permit (seasons ran annually from the Saturday nearest October 17^th^ until January 31^st^). We sent a follow up survey to all non-respondents and removed duplicate surveys. We sent surveys to a mean of 1220 (range = 165–2000) bobcat hunters/trappers annually, and annual response rates averaged 72.3% (range = 62.1%–78.3%) (Supporting Information 2). We asked hunters and trappers specific questions about their hunting and trapping methods used during the season (Supporting Information 2). From these surveys we calculated hunter and trapper participation, the number of days spent hunting (hunters) or trap-days (number of traps multiplied by number of days trapped; trappers), the percent of successful hunters and trappers, the number of bobcats released by hunters and trappers, and the number of bobcats chased by hound hunters. We calculated CPUE as animals harvested per day hunted/trapped and ACPUE as animals caught (both harvested and those captured and released) per day hunted/trapped. Of bobcat hunters/trappers who listed their method of take, 46.3% (annual range = 32.0–62.5%) hunted only with hounds, 25.4% (annual range = 15.2%–32.6%) only trapped, with others calling bobcats or using multiple methods (S1 Table). We excluded data from our analyses for hunters/trappers that used multiple harvest methods.

### Statistical analyses

We used program R version 3.3.1 [28] for all statistical analyses. We used generalized linear models (GLMs) to test for differences between successful and unsuccessful hunters/trappers for four dependent variables: the number of days hunted (hunters), the number of trap-days (trappers), and number of bobcats released (hunters and trappers). Because these dependent variables were count data, we used GLMs with quasi-Poisson error distributions and log links to correct for overdispersion. We also tested for correlations between the number of bobcats released by hunters or trappers and bobcat abundance.

We created CPUE and ACPUE metrics for hunters (reported as harvested bobcats per day and all bobcats caught per day) and trappers (reported as harvested bobcats per 100 trap-days and all bobcats caught per 100 trap-days). We calculated CPUE by dividing the number of bobcats harvested (0 or 1) by the number of days hunted or trapped. We then calculated ACPUE by summing bobcats caught and released with the bobcats harvested, then dividing by the number of days hunted or trapped. We created summary statistics for each variable and used a linear regression with Gaussian errors to determine if the metrics were correlated with year.

The relationship between CPUE and abundance generally follows a power relationship

$${\text{CPUE}}_{t}=\alpha N_{t}{}^{\beta }$$

where α is a catchability coefficient and β describes the shape of the relationship [9]. This formulation allows for non-linear relationships between CPUE and abundance (*N*) as well as linear relationships when β = 1.0. Values of β < 1.0 indicate hyperstability and values of β > 1.0 indicate hyperdepletion [9, 29]. Hyperstability implies that CPUE increases more quickly at relatively low abundances, perhaps due to increased efficiency or efficacy by hunters, whereas hyperdepletion implies that CPUE changes more quickly at relatively high abundances, perhaps due to the inaccessibility of portions of the population by hunters [30]. Taking the natural log of both sides creates the following relationship

$${\text{log}}_{e}\left({\text{CPUE}}_{t}\right)={\text{log}}_{e}\left(\alpha \right)+\beta \left[{\text{log}}_{e}\left(N_{t}\right)\right]$$

allowing one to test both the shape and strength of the relationship between CPUE and *N* [9, 29].

Because both the dependent and independent variables in this relationship are estimated with error, reduced major axis (RMA) regression may be used to provide less biased parameter estimates [31–33]. We used RMA to estimate the relationships between the log of CPUE and ACPUE for hunters and trappers and the log of bobcat abundance (*N*) using the *lmodel2* function in the R package lmodel2 [34]. Because RMA regressions may overestimate the strength of the relationship between CPUE and *N* when these variables are not correlated, we followed the approach of DeCesare et al. [30] and used Pearson’s correlation coefficients (*r*) to identify correlations between the natural logs of CPUE/ACPUE and *N*. We used α = 0.20 to identify correlated variables in these tests in order to limit Type II error due to small sample sizes. We divided each CPUE/ACPUE variable by its maximum value prior to taking their logs and running correlation tests [e.g., 30]. Bobcat abundance increased during 1993–2003 and decreased from 2005–2013 [27], and our preliminary analyses indicated that the relationship between CPUE and abundance varied over time as a function of the population trajectory (increasing or decreasing). We therefore estimated β for hunter and trapper CPUE separately during 1993–2002 and 2003–2013. We calibrated ACPUE using values during 2003–2013 for comparative purposes.

Finally, we evaluated the predictive ability of modeling CPUE and ACPUE as a function of annual hunter/trapper success (bobcats harvested/available permits) to assess the utility of hunter/trapper success for estimating CPUE/ACPUE for possible inclusion in population models when only hunter/trapper success is available. We first considered hunter metrics, then trapper metrics, and last considered an overall composite score using both hunter and trappers metrics. We calculated the composite score for year *t* and method *m* (hunter or trapper) as a weighted average of hunter and trapper success weighted by the proportion of harvest made by hunters and trappers as follows:

$$\left(w_{Hunter}{}_{,t}\right)\left(\text{CPUE}/{\text{ACPUE}}_{Hunter}{}_{,t}\right)+\left(w_{Trapper}{}_{,t}\right)\left(\text{CPUE}/{\text{ACPUE}}_{Trapper}{}_{,t}\right)$$

where *w*_*Hunter*,*t*_ + *w*_*Trapper*,*t*_ = 1. In each analysis we used linear regression with Gaussian errors, with the given hunter or trapper metric as our dependent variable, and success as our independent variables.

## Results

### Days hunted and trapped

**Fig 1:**
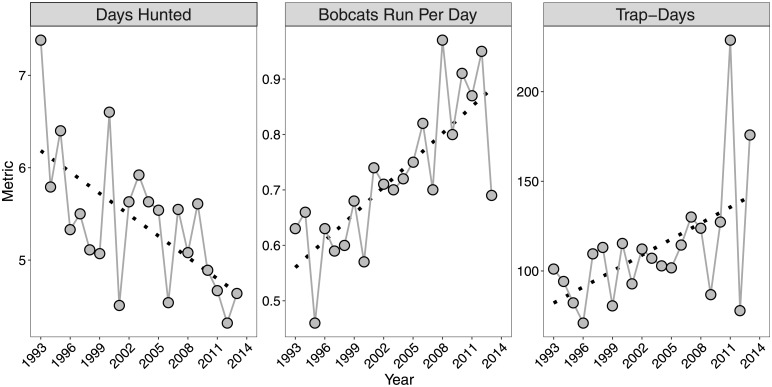
Annual variation in effort and success by bobcat hunters (days hunted and bobcats chased per day) and trappers (trap-days–number of days trapped multiplied by number of trap sets) in Wisconsin from 1993–2013. The dashed lines are estimated fits from linear regression models.

Hunters showed a decreasing trend in the number of days hunted over time (*r* = -0.63, *P* = 0.0020, Fig 1), but an increasing trend in the number of bobcats chased per day (*r* = 0.77, *P* < 0.0001, Fig 1). Contrary to our hypothesis, the number of days hunted did not differ between successful and unsuccessful hunters (${\bar{x}}_{\text{successful}}=5.69\pm 0.11$ SE; ${\bar{x}}_{\text{unsuccessful}}=5.48\pm 0.09$ SE; β = 0.04, *P* = 0.13).

Trappers exhibited substantial annual variation in the number of days trapped over time, but without a clear trend (*r* = -0.15, *P* = 0.52). Trappers who harvested a bobcat used more trap sets than trappers who did not (${\bar{x}}_{\text{successful}}=6.61\pm 0.25$ SE, ${\bar{x}}_{\text{unsuccessful}}=5.59\pm 0.12$ SE; β = 0.17, *P* < 0.01). The mean number of trap-days also showed an increasing trend (*r* = 0.52, *P* = 0.01, Fig 1). Trappers who harvested a bobcat had more trap-days ($\bar{x}=116.4\pm 6.0$ SE) than trappers who did not harvest a bobcat ($\bar{x}=103.5\pm 3.4$ SE) (β = 0.12, *P* = 0.04).

### Bobcats released

The mean number of bobcats released annually by hunters was 0.45 (range = 0.22–0.72) (Table 1) and showed no clear trend over time (*r* = -0.10, *P* = 0.76). Contrary to our hypothesis, there was no difference in the number of bobcats released between successful and unsuccessful hunters (successful: $\bar{x}=0.48\pm 0.04$ SE; unsuccessful: $\bar{x}=0.39\pm 0.05$ SE) (β = 0.20, *P* = 0.14). The annual number of bobcats released by hunters was not correlated with bobcat abundance (*r* = -0.14, *P* = 0.65).

**Table 1:** Annual estimates of various effort and harvest metrics by bobcat hunters and trappers in Wisconsin 1993–2013.

Year	Hunters					Trappers						
	Days Hunted	Success Rate	Catch Per Day	Mean Caught and Released	Actual Catch Per Day	Days in Field	Mean Sets	Trap-Days	Success Rate	Catch Per 100 Trap-Days	Mean Caught and Released	Actual Catch Per 100 Trap-Days
1993	7.38	22.5%	0.05	n/a	n/a	17.19	5.13	101.0	26.1%	0.80	n/a	n/a
1994	5.79	30.5%	0.07	n/a	n/a	18.35	4.95	94.1	17.9%	0.58	n/a	n/a
1995	6.40	22.9%	0.07	n/a	n/a	15.93	5.01	82.2	12.2%	0.50	n/a	n/a
1996	5.33	33.6%	0.11	n/a	n/a	15.79	4.69	71.0	16.7%	0.64	n/a	n/a
1997	5.50	20.3%	0.07	n/a	n/a	18.56	5.51	109.6	26.9%	0.70	n/a	n/a
1998	5.11	23.3%	0.07	n/a	n/a	18.47	5.46	113.2	20.4%	1.03	n/a	n/a
1999	5.07	32.1%	0.08	n/a	n/a	15.74	5.35	80.5	22.4%	1.20	n/a	n/a
2000	6.60	43.5%	0.12	n/a	n/a	19.97	5.33	115.3	24.2%	0.51	n/a	n/a
2001	4.51	36.3%	0.13	n/a	n/a	15.69	5.66	92.7	34.6%	1.60	n/a	n/a
2002	5.63	23.5%	0.07	0.56	0.16	18.06	6.20	112.3	36.9%	1.42	0.10	1.84
2003	5.92	47.5%	0.16	0.52	0.21	16.09	6.31	107.1	28.0%	1.77	0.17	2.08
2004	5.63	41.9%	0.15	0.45	0.21	16.88	6.06	102.9	37.1%	1.53	0.19	2.69
2005	5.54	58.3%	0.21	0.31	0.26	17.67	6.08	101.7	41.6%	1.87	0.11	2.08
2006	4.54	42.4%	0.17	0.25	0.21	17.87	6.29	114.4	50.0%	1.22	0.17	1.49
2007	5.55	70.8%	0.30	0.49	0.35	18.78	7.10	130.1	42.3%	3.01	0.19	3.40
2008	5.08	81.3%	0.32	0.51	0.41	18.15	5.77	123.8	51.4%	5.41	0.25	6.20
2009	5.61	68.6%	0.28	0.43	0.33	13.33	6.18	86.8	48.8%	2.39	0.26	2.93
2010	4.89	87.0%	0.42	0.72	0.54	16.85	7.72	127.3	60.0%	2.30	0.18	2.60
2011	4.67	72.1%	0.33	0.22	0.37	20.23	10.25	228.7	58.8%	1.45	0.52	1.87
2012	4.32	80.7%	0.39	0.54	0.45	12.50	6.53	77.8	60.0%	8.07	0.25	8.61
2013	4.64	83.8%	0.33	0.35	0.38	17.21	8.03	175.8	39.4%	6.02	0.15	7.93

Success rate is the proportion of licensed hunters/trappers that harvested a bobcat.

The mean number of bobcats released annually by trappers was 0.21 (range = 0.10–0.52) (Table 1) but was not correlated with year (*r* = 0.49, *P* = 0.11). Trappers who harvested a bobcat released more bobcats ($\bar{x}=0.37\pm 0.04$ SE) than trappers who did not harvest a bobcat ($\bar{x}=0.05\pm 0.01$ SE) (β = 2.04, *P* < 0.0001). The annual number of bobcats released by trappers was not correlated with bobcat abundance (*r* = -0.45, *P* = 0.15).

### Per-unit-effort metrics and abundance

**Fig 2:**
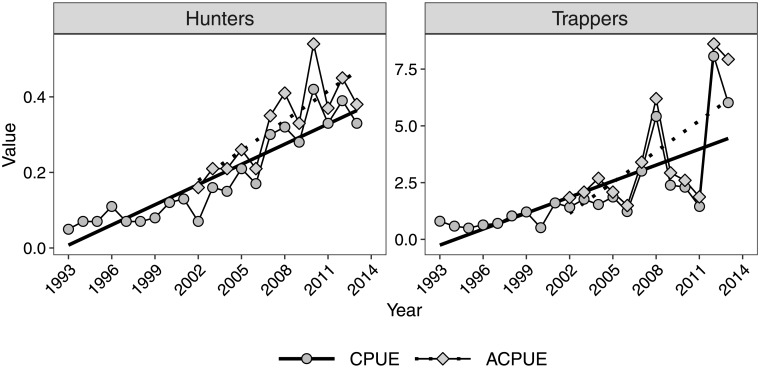
Annual catch-per-unit-effort (CPUE, bobcats per day) and actual catch-per-unit-effort (ACPUE, bobcats per 100 trap-days) estimates for bobcat hunters and trappers in Wisconsin during 1993–2013. Trend lines are the estimated fits from linear regression models fit separately for CPUE and ACPUE.

The mean CPUE was 0.19 bobcats/day for hunters (range = 0.05–0.42) and 2.10 bobcats/100 trap-days for trappers (range = 0.50–8.07) (Table 1). The mean ACPUE was 0.32 bobcats/day for hunters (range = 0.16–0.54) and 3.64 bobcats/100 trap-days for trappers (range = 1.49–8.61) (Table 1). The coefficient of variation for CPUE and ACPUE was greater for trappers than for hunters (trapper CPUE = 96%, hunter CPUE = 65%, trapper ACPUE = 68%, hunter ACPUE = 36%). All four metrics increased over time (Fig 2) although the strength of the relationship with year varied (hunter CPUE:, *r* = 0.92, *P* < 0.01; trapper CPUE: *r* = 0.73, *P* = < 0.01; hunter ACPUE: *r* = 0.82, *P* = < 0.01; trapper ACPUE: *r* = 0.66, *P* = 0.02).

**Fig 3:**
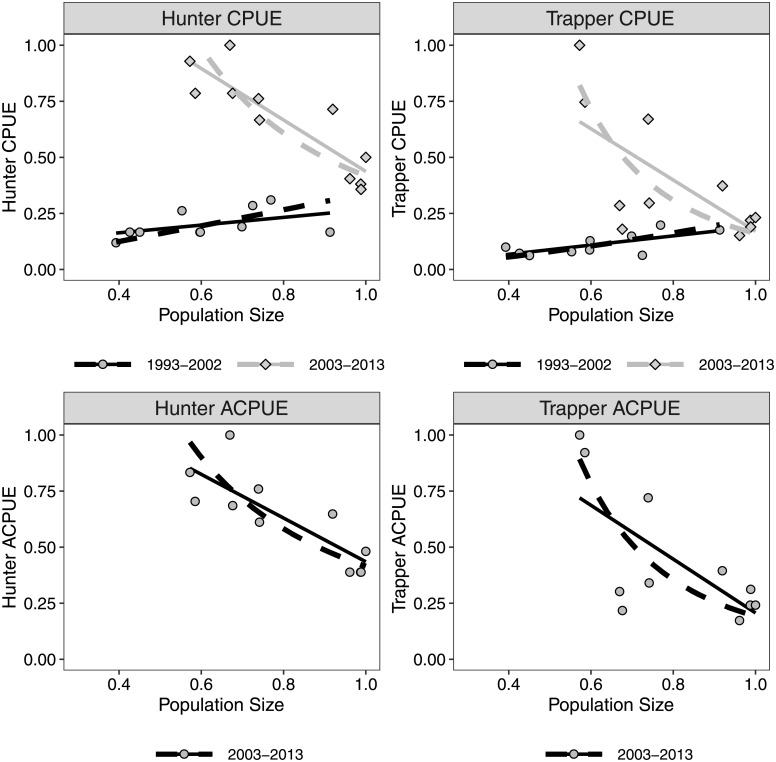
Estimated relationships between catch-per-unit-effort (CPUE) and actual catch-per-unit-effort (ACPUE) for bobcat hunters and trappers in Wisconsin and estimated bobcat abundance. Solid lines are estimated fits from linear regression models while dashed lines are estimated fits from reduced major axis regression of the log of CPUE/ACPUE against the log of abundance. The dependent and independent variables have been rescaled by dividing by the maximum value.

Hunter and trapper CPUE across all years was not correlated with bobcat abundance (*r* = 0.38, *P* = 0.09 and *r* = 0.32, *P* = 0.16, respectively). But during the two time periods we tested (1993–2002 and 2003–2014), the correlations between hunter and trapper CPUE and bobcat abundance were all correlated (|*r*| ≥ 0.63, *P* ≤ 0.05) except for hunter CPUE during 1993–2002 which had a marginal relationship (*r* = 0.54, *P* = 0.11, Table 2). The relationships between CPUE and abundance were positive during 1993–2002 although the 95% CI for β were wide and overlapped 1.0 for both hunter and trapper CPUE (Fig 3). The relationships between CPUE and abundance were negative during 2003–2014 and the 95% CI for β were < -1.0 indicating CPUE declined more rapidly at lower abundances (Fig 3). Hunter CPUE had the strongest relationship with bobcat abundance (R^2^ = 0.73, Table 2).

**Table 2:** Estimated parameters from reduced major axis regression of log of bobcat hunter and trapper catch-per-unit-effort (CPUE) and actual catch-per-unit-effort (ACPUE) against log of bobcat abundance (*N*) and Pearson’s correlation coefficient (*r*) and its test of significance. Estimates of β whose 95% CI include 1 or -1 indicate failure to reject the null hypothesis of a linear relationship between log(CPUE/ACPUE) and log(*N*) and are marked as bold.

Method	Metric	Period	α	β	95% CI (β)	R^2^	*R*	*P*
Hunter	CPUE	1993–2002	-1.08	1.09	**0.57–2.07**	0.29	0.539	0.11*
Hunter	CPUE	2003–2013	-0.87	-1.69	-2.47–-1.15	0.73	-0.856	<0.001
Trapper	CPUE	1993–2002	-1.47	1.54	**0.85–2.81**	0.39	0.625	0.05
Trapper	CPUE	2003–2013	-1.83	-2.92	-4.9–-1.74	0.48	-0.696	0.02
Hunter	ACPUE	2003–2013	-0.88	-1.52	-2.28–-1.01	0.69	-0.832	<0.001
Trapper	ACPUE	2003–2013	-1.65	-2.75	-4.65–-1.63	0.47	-0.686	0.02

ACPUE was negatively correlated with bobcat abundance for hunters (*r* = -0.83, *P* < 0.0001) and trappers (*r* = -0.69, *P* = 0.02). The 95% CI for β for the relationships between ACPUE and bobcat abundance were < -1for both hunter and trapper ACPUE although the relationship was stronger for hunter ACPUE (R^2^ = 0.69, Table 2).

### Modeling potential harvest and catch per-unit-effort

Annual hunter/trapper success was strongly related to both hunter CPUE (*F*_1,19_ = 505.4, R^2^ = 0.96, β = 0.61, *P* < 0.0001) and ACPUE (*F*_1,10_ = 101.2, R^2^ = 0.91, β = 0.68, *P* < 0.0001). Annual hunter/trapper success was also strongly related to trapper CPUE but with lower explanatory ability (*F*_1,19_ = 30.1, R^2^ = 0.61, β = 8.04, *P* < 0.0001) as was trapper ACPUE (*F*_1,10_ = 7.9, R^2^ = 0.44, β = 10.23, *P* = 0.02). We strongly predicted composite CPUE and ACPUE using annual hunter/trapper success (composite CPUE: *F*_1,19_ = 501.9, R^2^ = 0.96, β = 0.48, *P* < 0.0001; composite ACPUE: *F*_1,10_ = 111.6, R^2^ = 0.92, β = 0.56, *P* < 0.0001).

## Discussion

Per-unit-effort data can potentially provide valuable metrics both for understanding the role of harvest on wildlife population dynamics [4, 35, 36] and for estimating wildlife population trends, either directly or through inclusion in statistical population models [7, 8]. The relationship between CPUE and abundance in our study varied depending on the population trajectory, highlighting the importance of calibrating CPUE metrics prior to using them to evaluate population trends [1]. CPUE showed virtually no relationship with bobcat abundance across all years of our study, but both CPUE and ACPUE had stronger, non-linear, and negative relationships with abundance when the population was decreasing. Our results also illustrate the importance of testing for non-linear relationships between CPUE and abundance. Studies calibrating per-unit-effort metrics against abundance should also test for changes in the relationship between these variables during periods of different population trajectories (e.g., increasing or decreasing trajectories) and between different harvest methods (e.g., hunting and trapping).

In many instances per-unit-effort metrics are valuable indices for abundance, but they are not always cost effective to estimate. Despite the low costs of annual harvest questionnaires relative to mark-recapture or other field-intensive studies, annual questionnaires conducted over many years may still prove prohibitively expensive for some wildlife management agencies. We therefore tested simple models for estimating CPUE and ACPUE metrics from annual hunter/trapper success (bobcats harvested/available permits). We found that hunter/trapper success, generally an inexpensive metric that is readily available from harvest data without requiring annual questionnaires, can serve as a proxy for per-unit-effort metrics in population models for effective management and conservation. Hunter CPUE and ACPUE and our composite scores of CPUE and ACPUE were well predicted by hunter/trapper success (R^2^ > 0.9). However, the explanatory power of models for trapper ACPUE and CPUE was moderate (R^2^ ≤ 0.6). Nevertheless, our composite model was a strong fit for both CPUE and ACPUE and these values can easily be integrated into population models.

CPUE data may be easier and less expensive to collect over broad spatiotemporal extents than direct estimates of abundance but using CPUE as an index to directly monitor wildlife populations depends on the relationship between CPUE and abundance or density. While some studies have reported relatively strong, positive correlations between CPUE metrics and abundance or density [35, 37], others have reported more variable results [30, 36, 38, 39]. Hunter selectivity may help explain poor correlations between CPUE and abundance in species with selective or limited harvest [30, 39, 40]. ACPUE should account for hunter selectivity by including animals encountered but not harvested. However, we found similar or weaker relationships between ACPUE and abundance. This result was surprising because bobcat hunters in Wisconsin were more likely to harvest larger, older, and male bobcats for taxidermy mounts [13]. Hunters may therefore pass up opportunities to harvest less desirable individuals [e.g., 16] resulting in greater effort expended before harvesting an individual. It is possible that hunters/trappers re-encounter the same individual multiple times which may obscure the relationship between ACPUE and abundance, although we suspect this is unlikely for hunters given their ability to search a greater spatial area than trappers. The negative relationship we found with bobcat CPUE/ACPUE and abundance during the period of population decline, however, contrasts with predominately positive relationships between CPUE and abundance/density reported in previous studies of harvested terrestrial mammals [30, 37, 41, but see 36] and fish [9]. The nature of these relationships may also be affected by population trajectory, bag limit sizes, the role of trophy hunting, or hunter selectivity or experience [30, 41]. The accuracy of and uncertainty in the abundance estimates used in calibration is important as inaccurate or imprecise abundance estimates may further obscure calibration efforts. It is important to consider these effects on CPUE metrics in future studies, especially when using CPUE as an index of abundance.

Our estimates of the shape of the relationship between CPUE/ACPUE and bobcat abundance (i.e., our estimates of β) indicated primarily non-linear relationships suggesting that CPUE/ACPUE may not vary proportionally with abundance (i.e., β ≠ 1). CPUE showed virtually no relationship with bobcat abundance across all years, but a different pattern emerged when abundance was split into two time periods. When bobcat abundance was increasing CPUE showed a positive relationship not differing significantly from a linear relationship. However, when bobcat abundance was decreasing CPUE showed a significant non-linear negative relationship, especially for hunters, although we suggest caution in interpreting these results due to our small sample sizes. Bowyer et al. [38] also found a negative relationship between moose (*Alces alces*) harvest-per-unit-effort and abundance when abundance was low, but a positive relationship at higher abundances. CPUE metrics may also vary disproportionally with abundance or density if hunters are highly efficient at harvesting individuals or if certain segments of the population are unavailable for harvest [9, 42]. A significant non-linear negative relationship between CPUE/ACPUE and abundance, as seen when bobcat abundance was declining (i.e., β < -1), could indicate that CPUE/ACPUE exhibits a higher rate of change when abundance is small, analogous to hyperstability. Hyperstability can be caused by increased harvest efficiency [9, 30] which is consistent with our hypothesis that contemporary bobcat hunters and trappers are relatively motivated and skilled individuals with high participation and success rates despite decreasing bobcat abundance. Variable and/or non-linear relationships between CPUE/ACPUE may lead to misleading inferences regarding population trends but may also bias the results of statistical population reconstruction models which often assume β = 1 [8]. It is therefore important that wildlife managers thoroughly evaluate sources of variability in CPUE/ACPUE in addition to their relationships with abundance.

The strongest relationship between our per-unit-effort metrics and bobcat abundance was for hunter post-2002 CPUE and ACPUE, with weaker relationships for trappers. One hypothesis explaining the pattern for hunters is that declining permit availability has led to greater efficiency and success, which reduces the variation and uncertainty in our annual estimates. Bobcat permit availability has decreased and applicant numbers have increased in Wisconsin since approximately 2003 [23]. Bobcat hunters may therefore have increased their efficiency in order to maximize limited opportunities for bobcat harvest by hunting or trapping in the best available bobcat habitat or increasingly using the collective experience and knowledge of the bobcat hunter/trapper community. Consistent with this hypothesis, the proportion of permit holders annually participating in the bobcat hunt has increased from 55% in 1993 to 85% in 2013 [23]. Similarly, the highly restrictive permitting process may limit the applicant pool to relatively skilled and/or motivated individuals. For example, Ward et al. [32] found that lakes with low densities of larger rainbow trout (*Onchorhynchus mykiss*) attracted fewer but more experienced anglers resulting in increased catchability by individual anglers. We encourage additional research to test the hypothesis that greater harvest efficiency leads to reduced uncertainty in per-unit-effort metrics and stronger relationships with abundance. CPUE and ACPUE for trappers were less strongly correlated to bobcat abundance than for hunters. Trappers may show less selective harvest because of the difficulties of releasing a bobcat from a trap and/or because they put a greater emphasis on pelt sales than taxidermy mounts [13]. Trapper success was also affected by effort as successful trappers had more trap-days than unsuccessful trappers, and this relationship appeared driven by variation in number of traps sets rather than number of days in the field.

## Conclusions

Our results support the recommendations of previous studies to calibrate per-unit-effort metrics against abundance or density prior to their use for population monitoring [9, 41]. Importantly, our results show that the relationships between these metrics and abundance can vary depending on the trajectory of the population and may be relatively insensitive to changes in abundance. This may be particularly important for species with restricted and/or selective harvest. While ACPUE incorporates individuals captured but not harvested and may therefore be more ideal than CPUE in limited-harvest systems, we found that ACPUE and CPUE had generally similar relationships to abundance. The relationships between CPUE/ACPUE and harvest success shows promise for estimating per-unit-effort metrics from relatively simple and inexpensive data sources (e.g., annual harvest divided by the number of available permits). Given the potential applicability of per-unit-effort metrics for monitoring populations via statistical modeling we encourage additional study-specific efforts for both calibrating and estimating per-unit-effort metrics, particularly in populations with temporally-varying population trends.

## Supporting information

S1 File An example of the surveys sent by the Wisconsin department of natural resources to every bobcat hunter/trapper annually (2012 survey used as an example). (PDF)

S1 Table The results of our bobcat hunter/trapper surveys during 1993–2013. We report the number of surveys sent, number of responses received and the response rate. We then report the number of hunters/trappers in each category of harvest method. In our analyses we excluded data from hunters/trappers that used multiple harvest methods in order to use data that was exclusively from hunters or trappers.
(DOCX)
