# Supplementary material for: Relationships of catch-per-unit-effort metrics with abundance vary depending on sampling method and population trajectory
Source: PLoS One. 2020 May 21;15(5):e0233444. doi: 10.1371/journal.pone.0233444 (PMC7241727; doi:10.1371/journal.pone.0233444)
Supplement: S1 File — (PDF) [file pone.0233444.s001.pdf]

Supplementary Material 1. An example of the 2012 annual bobcat hunter survey from the Wisconsin Department of Natural Resources. Annual surveys are available from: https://dnr.wi.gov/topic/wildlifehabitat/reports.html

State of Wisconsin
Department of Natural Resources

Wisconsin Bobcat Hunting / Trapping Questionnaire

s. 23.09(2)(k), Wis. Stats.
Form 2300-232 (R 11/12)

Dear Bobcat Hunter/Trapper:

You have been selected to help the Department of Natural Resources gather much needed information on Wisconsin's bobcat harvest. Please help our furbearer management program by completing this voluntary questionnaire. We would like your input regarding bobcat season and management.

Using your stamp will save funds for management, even though no postage is required to return the questionnaire. Your time and effort is greatly appreciated. Thank you!

Sincerely,

John F. Olson

John F. Olson
Furbearer Ecologist
Bureau of Wildlife Management

1. Did you hunt and/or trap bobcat during the 2012-2013 season?
   (1) ☐ Yes (2) ☐ No

2. If no, why didn't you hunt and/or trap for bobcat?
   (1) ☐ Poor weather (2) ☐ Too far to travel
   (3) ☐ Other (please explain): ____________

3. Knowing it may require five years or more of continuous application, will you continue to apply for a bobcat permit in the future?
   (1) ☐ Yes (2) ☐ No

4. Overall, what was your impression of the "split" bobcat season?

No Opinion	Did Not Like It						Did Like It
0	1	2	3	4	5	6	7

IF YOU DID NOT HUNT OR TRAP, STOP HERE AND RETURN THIS QUESTIONNAIRE. THANK YOU!

5. What primary harvest technique did you use?
   (1) ☐ Trapping (2) ☐ Predator Calls (3) ☐ Hunting Dogs

6. Did you register a bobcat during the 2012-2013 season (October 20 - December 25 or December 26 - January 31) ?
   (1) ☐ Yes (2) ☐ No

6a. If yes, what was its' final disposition?
   (1) ☐ Sold for the fur trade (3) ☐ Tanned and kept or sold
   (2) ☐ Made into a taxidermy mount

7. How would you rate the hunting/trapping conditions during **your** bobcat season?

1	2	3	4	5	6	7
Very Poor			Average			Perfect

Please rate the following factors and how they may have influenced your bobcat hunting/trapping:

	No Impact	Negative Impact				Positive Impact
Cold Weather	0	1	2	3	4	5
Presence of tracking snow (1" - 2")	0	1	2	3	4	5
Walking snow (2"+ to 12")	0	1	2	3	4	5
Snowshoe snow (12"+)	0	1	2	3	4	5
Impassable roads (snowed in)	0	1	2	3	4	5
Split Season decreased the number of permits in the field	0	1	2	3	4	5
Other bobcat hunters/trappers	0	1	2	3	4	5
Other forest users	0	1	2	3	4	5

8. If you **TRAPPED** bobcat during the 2012-2013 season, please answer the following questions:
   Number of days trapped ____________
   Average number of sets/day ____________
   Number of bobcats located ____________

9. Type of sets used:
   ____________# of foothold sets made
   ____________# of body-gripper sets
   ____________# of cage sets
   ____________# of cable restraint sets made

10. Did you release any bobcats from your traps during the 2012-2013 season?
    (1) ☐ Yes (2) ☐ No
    If yes, how many? ____________

11. If you HUNTED bobcat during the 2012-2013 season, please answer the following questions:
    Number of days hunted with dogs ____________
    Number of days hunted without dogs ____________
    Number of bobcats run with dogs ____________
    Number of bobcats located ____________
    Number of days hunted using predator calls ____________

Figure 1. *The 2012-2013 Wisconsin bobcat hunting/trapping questionnaire.*

12. Did you pass on any treed bobcats during the 2012-2013 season?

(1) ☐ Yes (2) ☐ No If yes, how many? ______________

13. In your bobcat hunting/trapping efforts, did you: (Check all that apply)

(1) ☐ Hunt/trap alone
(2) ☐ Hunt/trap with friend(s)
(3) ☐ Participate in a guided trip (go to 13a)

13a. If you participated in a guided trip, generally what was the fee?

(1) ☐ less than $500
(2) ☐ $500 to $999
(3) ☐ $1,000 to $1,499
(2) ☐ $1,500 to $1,999
(3) ☐ $2,000+

14. In which counties did you hunt and/or trap bobcats? (Please list the county you spent the most time in first.)

______________ ______________
______________ ______________

15. Have you observed bobcats SOUTH of the current hunting zone (current zone is all of WI north of Hwy 64)?

(1) ☐ Yes (2) ☐ No

15a. If yes, please fill in the following information:

# of Road Kill	County	# of Live Sitings	County

16. In your opinion, how does the current bobcat, fox, coyote, fisher, and gray wolf population compare to last year? (Check one for each species)

	Bobcat	Fox	Coyote	Fisher	Gray Wolf
More abundant than last year	☐	☐	☐	☐	☐
Less abundant than last year	☐	☐	☐	☐	☐
About the same as last year	☐	☐	☐	☐	☐
No opinion	☐	☐	☐	☐	☐

16a. In your opinion, how does the current population of these same furbearers compare to ten years ago? (Check one for each species)

	Bobcat	Fox	Coyote	Fisher	Gray Wolf
More abundant than ten years ago	☐	☐	☐	☐	☐
Less abundant than ten years ago	☐	☐	☐	☐	☐
About the same as ten years ago	☐	☐	☐	☐	☐
No opinion	☐	☐	☐	☐	☐

17. In which habitat type did you hunt and/or trap for bobcat the most? (Check one)

Upland Conifers	Upland Hardwoods	Lowland Forest / Swamp
(1) ☐ Regeneration / Brush	(1) ☐ Regeneration / Brush	(1) ☐ Regeneration / Brush
(2) ☐ Thinned / Pole-sized	(2) ☐ Thinned / Pole-sized	(2) ☐ Thinned / Pole-sized
(3) ☐ Large / Mature	(3) ☐ Large / Mature	(3) ☐ Large / Mature

18. Did you experience any competition or over-crowding with other bobcat hunters?

(1) ☐ Yes (2) ☐ No

If yes, what county or counties did you experience competition or over-crowding with other bobcat hunters?

____
____
____

19. Did you experience any competition or over-crowding with other bobcat trappers?

(1) ☐ Yes (2) ☐ No

If yes, what county or counties did you experience competition or over-crowding with other bobcat trappers?

____
____
____

20. Did you experience any competition or over-crowding with the outdoor activities of others?

(1) ☐ Yes (2) ☐ No

If yes, what county or counties did you experience competition or over-crowding?

____
____
____

Please add any comments you would like to offer regarding bobcat populations, harvest regulations or overall bobcat management.

Figure 1. *Continued.*
