# Supplementary material for: Relationships of catch-per-unit-effort metrics with abundance vary depending on sampling method and population trajectory
Source: PLoS One. 2020 May 21;15(5):e0233444. doi: 10.1371/journal.pone.0233444 (PMC7241727; doi:10.1371/journal.pone.0233444)
Supplement: S1 Table — We report the number of surveys sent, number of responses received and the response rate. We then report the number of hunters/trappers in each category of harvest method. In our analyses we excluded data from hunters/trappers that used multiple harvest methods in order to use data that was exclusively from hunters or trappers. (DOCX) [file pone.0233444.s002.docx]

**Supporting Information 2**

**Table S1. The results of our bobcat hunter/trapper surveys from 1993–2013.**

|  | **Surveys** | | |  | **Method of Harvest** | | | | |
| --- | --- | --- | --- | --- | --- | --- | --- | --- | --- |
| **Year** | **# of surveys sent** | **# of Responses** | **Response rate** |  | **Only Hound** | **Only Trap** | **Other** | **Multiple Methods** | **Unknown Methods** |
| 1993 | 2000 | 1393 | 69.7% |  | 280 | 199 | 204 | 56 | 20 |
| 1994 | 2000 | 1491 | 74.6% |  | 272 | 211 | 220 | 71 | 52 |
| 1995 | 2000 | 1478 | 73.9% |  | 258 | 208 | 222 | 82 | 33 |
| 1996 | 2000 | 1472 | 73.6% |  | 254 | 206 | 204 | 78 | 38 |
| 1997 | 2000 | 1486 | 74.3% |  | 311 | 286 | 187 | 92 | 96 |
| 1998 | 1860 | 1457 | 78.3% |  | 332 | 225 | 165 | 87 | 22 |
| 1999 | 1540 | 1166 | 75.7% |  | 292 | 198 | 122 | 73 | 9 |
| 2000 | 1490 | 1167 | 78.3% |  | 333 | 196 | 105 | 81 | 12 |
| 2001 | 781 | 485 | 62.1% |  | 150 | 81 | 40 | 41 | 7 |
| 2002 | 1330 | 993 | 74.7% |  | 276 | 222 | 60 | 80 | 42 |
| 2003 | 1379 | 957 | 69.4% |  | 343 | 189 | 61 | 94 | 45 |
| 2004 | 1352 | 954 | 70.6% |  | 358 | 187 | 65 | 100 | 40 |
| 2005 | 1541 | 1087 | 70.5% |  | 378 | 190 | 55 | 118 | 65 |
| 2006 | 1000 | 718 | 71.8% |  | 246 | 149 | 19 | 70 | 44 |
| 2007 | 1030 | 704 | 68.3% |  | 318 | 131 | 22 | 77 | 16 |
| 2008 | 540 | 394 | 73.0% |  | 196 | 72 | 3 | 50 | 11 |
| 2009 | 477 | 342 | 71.7% |  | 177 | 43 | 8 | 39 | 16 |
| 2010 | 455 | 333 | 73.2% |  | 161 | 65 | 14 | 31 | 3 |
| 2011 | 475 | 330 | 69.5% |  | 149 | 81 | 17 | 40 | 0 |
| 2012 | 165 | 115 | 69.7% |  | 57 | 20 | 5 | 20 | 0 |
| 2013 | 215 | 161 | 74.9% |  | 80 | 34 | 6 | 17 | 0 |

We report the number of surveys sent, number of responses received and the response rate. We then report the number of hunters/trappers in each category of harvest method. In our analyses we excluded data from hunters/trappers that used multiple harvest methods in order to use data that was exclusively from hunters or trappers.
